# Distribution of carbon, nitrogen and phosphorus in coastal wetland soil related land use in the Modern Yellow River Delta

**DOI:** 10.1038/srep37940

**Published:** 2016-11-28

**Authors:** Junbao Yu, Chao Zhan, Yunzhao Li, Di Zhou, Yuqin Fu, Xiaojing Chu, Qinghui Xing, Guangxuan Han, Guangmei Wang, Bo Guan, Qing Wang

**Affiliations:** 1 College of Resource and Environmental Engineering, Ludong University, Yantai 264025, P. R. China; 2 Key Laboratory of Coastal Environment Processes and Ecological Remediation, Yantai Institute of Coastal Zone Research, Chinese Academy of Sciences, Yantai 264003, P. R. China; 3 University of Chinese Academy of Sciences, Beijing 100049, P. R. China

## Abstract

The delivery and distribution of nutrients in coastal wetland ecosystems is much related to the land use. The spatial variations of TOC, TN, NH_4_^+^-N, NO_3_^−^-N and TP and associated soil salinity with depth in 9 kinds land uses in coastal zone of the modern Yellow River Delta (YRD) was evaluated based on monitoring data in field from 2009 to 2015. The results showed that the average contents of soil TOC, TN, NO_3_^−^-N, NH_4_^+^-N and TP were 4.21 ± 2.40 g kg^−1^, 375.91 ± 213.44, 5.36 ± 9.59 and 7.20 ± 5.58 and 591.27 ± 91.16 mg kg^−1^, respectively. The high N and C contents were found in cropland in southern part and low values in natural wetland, while TP was relatively stable both in profiles and in different land uses. The land use, land formation age and salinity were important factors influencing distributions of TOC and N. Higher contents of TOC and N were observed in older formation age lands in whole study region, while the opposite regulation were found in new-born natural wetland, indicating that the anthropogenic activities could greatly alter the original distribution regulations of nutrients in coastal natural wetlands by changing the regional land use.

The crucial role of nutrients in aquatic ecosystems has been widely acknowledged and has individually received great attention worldwide^1^,^2^. Especially the biogeochemical cycles and transport of carbon (C), nitrogen (N), and phosphorus (P) in coastal area have become a noticeable concern and interesting issue during the recent decades^1^,^3^,^4^,^5^,^6^. The coastal wetland system is an important link between estuaries and the coastal waters. Under the functions of land and ocean interactions, the biogeochemical processes transform nutrients ultimately controlling the quantity and distribution of carbon, nitrogen, and phosphorus in coastal wetlands^1^,^6^,^7^,^8^,^9^.

The coastal wetlands are regarded as most vulnerable aquatic ecosystems to climate change and anthropogenic impacts because of seawater intrusion, sea level rise, especially rapid population growth and uncontrolled development in some coastal regions worldwide. Based on the report by Kennish^10^, approximately four billion people live in coastal areas and this number is estimated to six billion by 2025. The estuaries will be most significantly impacted by habitat loss and alteration associated with a burgeoning coastal population. About two-thirds of the original coastal wetlands have been lost since European settlement, and the remaining 126,000 ha of U.S. coastal wetlands and ≥ 70,000 ha of Canadian wetlands are affected by anthropogenic stressors^11^. In China, there are about 15% population live coastal zone, which is only about 2.9% total area. About 50% of China’s coastal wetland was lost during 1949–2000 because of anthropogenic activities^12^. According to our results of remote sensing interpretation and field survey, about 204 km^2^ natural coastal wetland of Yellow River delta (YRD) is lost during 2000–2009 and most of which is transformed to farmland and aquaculture pond^13^. The land use is much related to the delivery and distribution of nutrients such as C, N and P in ecosystems^14^,^15^,^16^. Approximately, a third carbon emissions brought by land use change caused the reduction of soil organic matter content^17^.

Previous studies in YRD have reported the soil organic carbon (SOC) budget^13^, the distribution characteristics of SOC and nutrients stoichiometry in new-born coastal wetlands^18^,^19^, the nutrients exchange between land and atmosphere^20^,^21^,^22^. However, whole ecosystem scale nutrients information regarding the biogeochemical processes of C, N and P responsible for regulating the spatial distribution related land use and salinity studies in the YRD coastal systems is still lacking. Herein we report on nutrients of total organic carbon (TOC), total nitrogen (TN), ammonium (NH_4_^+^-N), Nitrate nitrogen (NO_3_^−^-N) and total phosphorus (TP) measured in soils at several coastal wetland sites in the whole modern YRD and assess the effects of land use classifications and soil salinity properties on nutrient distributions. The objectives of the present study were to determine (1) the spatial distribution of TOC, TN, NH_4_^+^-N, NO_3_^−^-N and TP related with land use; (2) the relations of soil nutrient distributions and anthropogenic activities and studied region formation period, and (3) the effects of soil salinity on nutrients in coastal region.

## Materials and Methods

### Description of the study area

**Figure 1:**
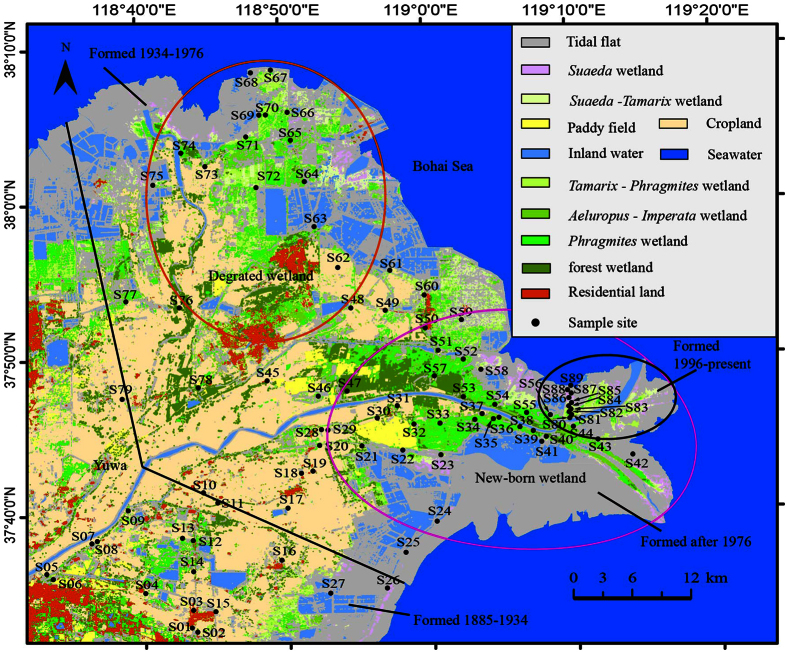
The land use and sample sites in the Modern Yellow River Delta (ARCGIS 9.3, http://www.esri.com).

The YRD (36°55′–38°16′N, 117°31′–119°18′E) is located in middle of eastern China, the southern coast of the Bohai Gulf and the western Laizhou Bay with an area of approximately 5400 km^2^ (shown in Yu, *et al*. 2012b, Fig.1a). The coastal wetland of the YRD is the most efficiently conserved, broadest and youngest wetland ecosystem in warm temperature zone in China^23^. Since it is in the sensitive areas to land-ocean interaction, a large number of wetland is formed because of rapid sediment accumulation from land runoff at estuary. Currently, there are series ecological problems of uncontrolled land reclamation, road and dam construction, river dry-up, pollution, sea level rise and coastal erosion^24^,^25^, leading to the coastal wetlands decline seriously and the ecosystems degraded gradually^26^. Most of land use is changed from natural wetlands to farm land, saltern-culture pone which close related human being activities^13^. In the study, the modern YRD (37°26′–38°09′N, 118°33′–119°18′E) (Fig. 1), which was formed since the watercourse of the Yellow River changed in 1855, was selected to evaluate the distributions of C, N and P because the land use change which were related with the anthropogenic activities was gradually elevated since then. The climate of the region belongs to warm temperate continental monsoon climate. The annual average temperature is 11.7–12.8 °C and the annual average rainfall is 530–630 mm, which of 70% is in the summer. The evaporation is 1900–2400 mm, and the drought index is up to 3.56^27^.

The dominant soil types are classified as Calcaric Fluvisols, Gleyic Solonchaks and Salic Fluvisols (FAO), which developed on loess material carried by water from the Loess Plateau. More than 85% species of the natural vegetation are salt tolerant plants and aquatic plants. The predominant species are *Phragmites australis, Suaeda heteropter Kitag, Aeluropus sinenis, Tamarix chinensis Lour*. and *Imperata cylindrica (Linn.) Beauv*^28^.

### Sampling and analytical methods

Landsat Thematic Mapper (TM) digital images with ground resolution of 30 meters of 2009 were used to study the lands use and land cover in the study region. The methods of Jensen *et al*.^29^ and Lavery *et al*.^30^ were used to remove radiometric and atmospheric effects by subtracting the radiance of a “dark pixel” within each band image. With the field investigation calibration, the land use map of the modern YRD was produced using supervised maximum likelihood classification from TM data (Fig. 1).

According to grid distribution point method, A total of 89 soil sampling sites including ten (S80-S89) of them in a monitoring transect (Fig. 1). Soil samples (three replicates) of 0–30 cm soil depth in 79 soil sites (S01-S79) and 0–60 cm soil depth in ten soil sites of each transect (total two parallel transects) were collected in the modern YRD during 2009–2015 (Fig. 1B). The soils from sites of S01-S79 were sampled in August (vigorous plants growth period) each year and those from sites of S80-S89 were sampled in each month from May to October (plant growth season) in study period. In each site, soil samples from different depths with 10 cm intervals were collected in soil profile. A total of 20 097 soil samples were collected. The air dry soil samples were kept no more than 2 weeks in sealed plastic bags at 5 °C to limit the microorganism activities until sieved through a 2 mm coarse stainless steel sieve for nutrients analysis. Roots and other organic matters were removed before analysis. Soil TOC was determined using Total Organic Carbon Analyzer (TOC-V_CPH_, Shimadzu, Japan). Contents of TN, NH_4_^+^-N, NO_3_^−^-N and TP were determined by Continuous Flow Analyzer (SKALAR-SAN^++^, Netherland). Soil pH and EC values were measured with electricity conduction method (soil/water = 1:5).

## Results

### The land use in modern Yellow River

**Figure 2:**
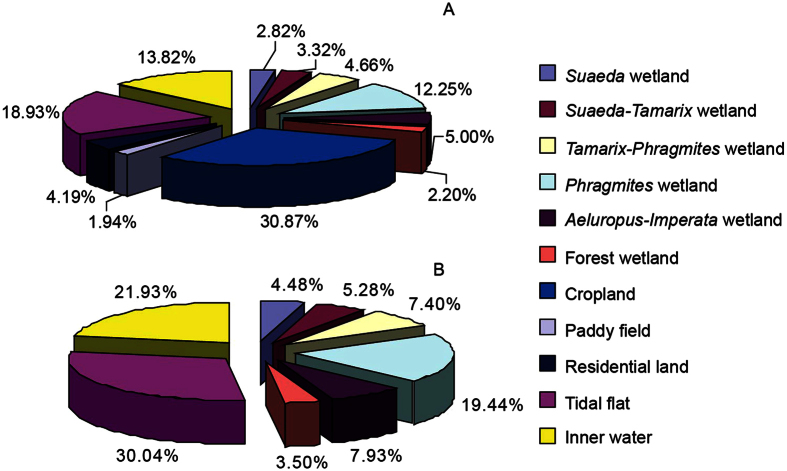
The area percentages of land use (**A**) and natural wetland (**B**) in Yellow River Delta.

Based on the interpretation results of TM digital images of 2009 and field investigation, the land use types in YRD were divided into natural wetlands of tidal flat, *Suaeda* wetland, *Suaeda-Tamarix* wetland, *Tamarix-Phragmites* wetland, *Aeluropus-Imperata* wetland, *Phragmites* wetland, forest wetland and inner water (water body), constructed wetlands of paddy field and saltern-culture pone, cropland (cotton is dominant) and residential land. More than 30% of total area is cropland (Fig. 2A) which appears in the middle of the YRD (Fig. 1). The area of natural wetlands, which mainly distribute close to the border of seawater, is about 63.18% of total area. Tidal flat is biggest natural wetland types in the region (Fig. 2B). Besides tidal flat and inner water, there are about 26.84% of natural wetlands belongs to the *Phragmites* wetland and *Tamarix-Phragmites* wetland. Therefore the *Phragmites australis* is predominant natural plant species in the coastal wetland of the YRD. Only small area of forest wetland (about 3.50% of total area of natural wetland) distributes in study region. Based on formation period, The modern YRD can be divided four parts, i.e. 1855–1934, 1834–1976, 1976–1996 and 1996-present (Fig. 1). More than 80% land use in area which formed during 1855–1934 has been changed to crop and residential land. The dominant land use in 1834–1976 formation area includes cropland, natural wetland and tidal flat. The land uses of the natural wetland and tidal flat mainly distribute in region which formed in 1976–1996 and 1996-present, respectively. Since 1976 when the watercourse of Yellow River changed from north into east direction, the natural wetland in north part began to degrade because of short of the fresh water supply and seawater intrusion. Thus the natural wetland types in the modern YRD can be divided into degraded wetland (485 km^2^) which distributed in north part and new-born wetland (1 045 km^2^) which located in east part (Fig. 1).

### The contents of soil C, N and P in coastal wetland

The range of soil TOC content in 0–30 cm soil layer in study region was 0.69–20.23 g kg^−1^ with average value of 4.21 ± 2.40 g kg^−1^ (Table 1). The average contents of TN, NO_3_^−^-N and NH_4_^+^-N in 0–30 cm soil layer were 375.91 ± 213.44, 5.36 ± 9.59 and 7.20 ± 5.58 mg kg^−1^. The range of soil TP content in study region was 281.09–1023.41 mg kg^−1^ with average value of 591.27 ± 91.16 mg kg^−1^ and median value of 579.64 mg kg^−1^.

The soil nutrient contents of TOC and N decreased with soil depth in 0–30 cm soil profiles (Table 1). The average contents of both TOC and NO_3_^−^-N in topsoil (0–10 cm) were about 1.6 times of those in 20–30 cm soil layer. The median values of N, especially NO_3_^−^-N, were much lower than their corresponding average values in different soil depth layer, indicated that N contents in several land uses were much higher than others. The big range of C and N contents with high standard deviation revealed that there was an obvious difference of nutrient distribution in the land uses.

### The spatial distribution of C, N and P in different land uses

**Figure 3:**
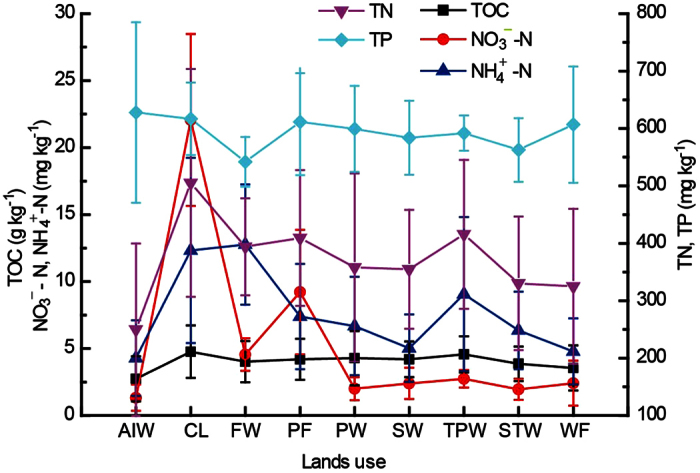
The nutrient contents in different land use soils of the modern Yellow River Delta (AIW, *Aeluropus-impeata* wetland, *n* = 2331; CL, Cropland, *n* = 1323; FW, Forest wetland, *n* = 189; PF, Paddy field, *n* = 189; PW, *Phragmites* wetland, *n* = 3339; SW, *Sueada* wetland, *n* = 1134; TPW, *Tararix-Phragmites* wetland, *n* = 567; STW, *Sueada-Tararix* wetland, *n* = 1008; WF, Tidal flat, *n* = 2457), vertical bar stands for standard deviation.

The 0–30 cm soil nutrient contents in nine land use types besides inner water and residential land in the modern YRD were shown in Fig. 3. The peak of average TOC content of 4.76 g kg^−1^ appeared in soils of cropland, followed with paddy field (4.20 ± 1.53 g kg^−1^). The soil NO_3_^−^-N contents in cropland and paddy field were much higher than that in the natural wetlands (Fig. 3). The range of average NO_3_^−^-N contents in natural wetlands was 1.33–4.55 mg kg^−1^, which was about 6%-20% of that in cropland. The high contents of NH_4_^+^-N were observed in cropland (12.32 mg kg^−1^) and forest wetland (12.76 mg kg^−1^). The average TN content in cropland was 505.28 mg kg^−1^, which was approximately 100 mg kg^−1^ higher than those in other land use. Besides of forest wetland, the difference of average TP contents in different land use was not obvious (Fig. 3).

**Figure 4:**
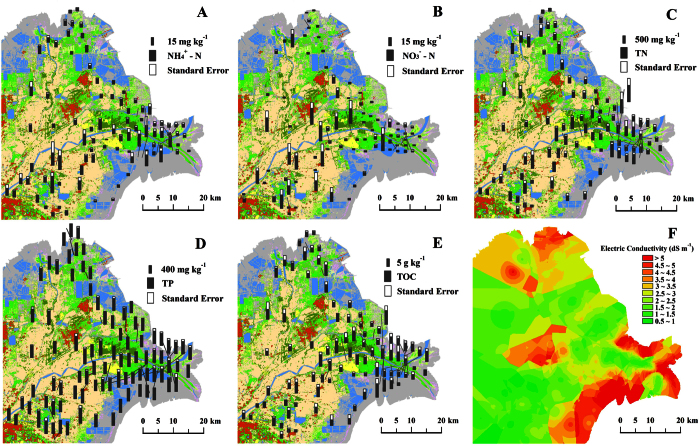
The distribution of NH_4_^+^-N (**A**), NO_3_^−^-N (**B**), TN (**C**), TP (**D**), TOC (**E**) and salinity (**F**) in the modern Yellow River Delta (ARCGIS 9.3, http://www.esri.com).

**Figure 5:**
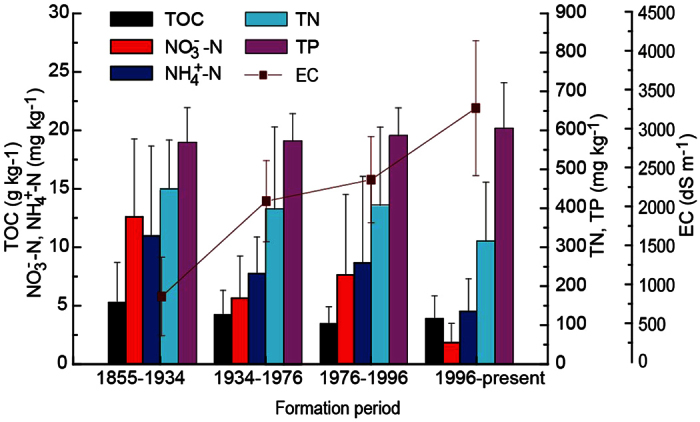
The distribution of soil nutrients in land uses of different formation period, vertical bar stands for standard deviation.

The spatial distribution characteristics of C, N and P were different in northern, southern and eastern parts of the YRD (Fig. 4A–E). The high soil contents of NH_4_^+^-N and NO_3_^−^-N were observed in southern part of YRD (Fig. 4A,B). TN contents in most of sample sites in southern part and eastern part were higher than those in northern part (Fig. 4C). The difference of soil P contents in different parts of the YRD was relatively small (Fig. 4D). Although little of vegetation grew on the tidal flat, the soil TOC contents there were not much lower than those in the middle of the YRD (Fig. 4E). Further analysis, we found that the average contents of TOC, NH_4_^+^-N, and NO_3_^−^-N in land uses which formatted during 1855–1934 were higher than those in other land uses (Fig. 5). The average TN in land uses of new-born wetland (formatted 1996-present) was 315.47 ± 150.36 mg kg^−1^, which was lower than those in soils of formation period of 1855–1934, 1934–1976 and 1976–1996. The significant differences of TP in four formation period soils were not observed (*p* ≥ 0.05).

## Discussions

### The nutrients contents of wetlands

The present results of soil TOC content (0.69–20.23 g kg^−1^) in the YRD (Table 1) were similar to the most of coastal wetlands of China^18^,^19^,^31^,^32^,^33^,^34^,^35^ and Sundarban mangrove wetland^36^ and the Mai Po Marshes coastal wetland^37^, but much lower than that in Louisiana coastal wetlands^38^. Compared to the reported results of coastal wetlands, the soil N contents in present study were much lower than those in Yangtze Estuary (southern China)^33^,^39^,^40^ and coastal wetlands of southern India^41^. In contrast, the very high soil TP contents were observed in study region, even about 2 times of that in marsh soils of river marginal wetlands^33^,^39^,^42^. The nutrient characteristics of YRD were much related to its formation processes. The YRD was formed by functions of alluvium and siltation. Large amounts of sediment joined into water when Yellow River flowed through the Loess Plateau. In the backwater effect of seawater, the flow rate of river water became slow and large proportion of sediment in water was deposited quickly at estuary^43^,^44^. During long distance transportation, most of the soluble fractions of nutrients such as NH_4_^+^-N, NO_3_^−^-N in sediment were lost, while insoluble components including P were kept in sediments and deposited at estuary.

**Figure 6:**
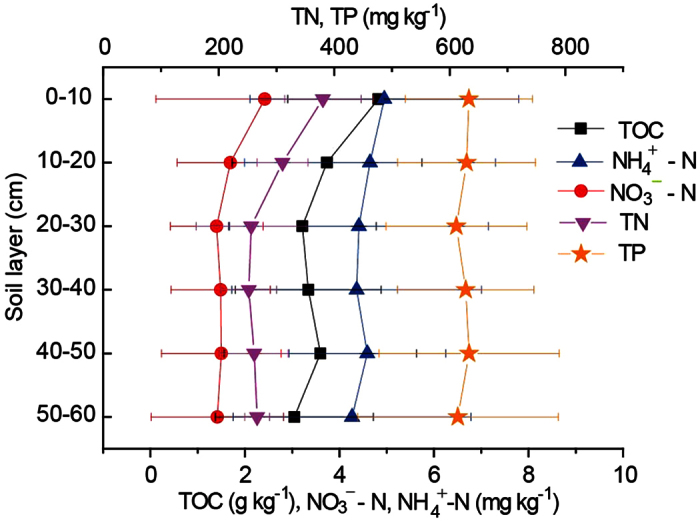
The vertical variations of nutrients in soil profiles of new-born wetland in Yellow River Delta (S80-S89), vertical bar stands for standard deviation.

Similar to the most of wetland ecosystems^34^,^39^,^45^,^46^, the high nutrient contents of N and C were observed in topsoil and decreased with depth in soil profiles under functions of vegetation enrichment (Table 1, Fig. 6). The vegetation could remarkably alter the vertical distribution of nutrients by changing the surrounding environment such as soil moisture, pH value and soil mechanical components^47^. Although the TP content in 0–10 cm soil layer is slight higher than that in 10–30 cm, TP showed the distinct vertical variation pattern with N in the soil profile, with stable contents of 610 g kg^−1^–635 g kg^−1^. The significant relation of TP and TOC in soil profile was not observed in the study. The previous studies demonstrated that organic P in the coastal sediment of the estuary was less than 20%^39^,^48^ and the distribution and accumulation of P in the estuary was independent of the grain size and the organic matter of the sediment^39^.

### Land use and land formation age

The soil properties including nutrients distribution and budgets are greatly related with land use^7^,^49^ and the land use changes was much influenced by anthropogenic activities which might cause a shift in the composition of nutrients and chemical processes^16^,^50^,^51^,^52^. Since 1855, about 37% of total area of the YRD has been changed from natural wetland to cropland and paddy field (Fig. 2A), which mainly formed during 1855–1976 and distributed in middle of study region (Fig. 1). Long-term experimental studies confirmed that the land use changed from native ecosystems to agriculture systems should result in loss of organic carbon^53^,^54^. However, we found the average values of TOC, TN, NO_3_^−^-N and NH_4_^+^-N in cropland which mainly distributed in middle of study region were much high than that in native ecosystems (Figs 3 and 4). The major sources of N and C for cropland soil were organic matter and fertilizer input. The dominated crop in the YRD is cotton because of its high soil salinity tolerance. Based on our survey results, about 1500–2000 kg ha^−1^ organic fertilizer and 10–16 kg N ha^−1^ were applied to keep the cotton yield, resulting in the high soil C and N in middle part. It is easy to understand that the great impact of human activities in the early formatted land, leading to the ratio crop land in different study part increasing with formation age. Hence the TN and TOC content tend to decrease with decreasing land formation age from 1855 to present and from 1855 to 1996, respectively (Fig. 5). The soil TOC in the new-born wetland (formatted at period of 1996-present) was similar to that in formation period of 1934–1976, which higher than that in formation period of 1976–1996 (Figs 4E and 5), indicating there was a special C source besides plant decomposition for the natural coastal wetlands. It is believed that this should be mainly from materials by tide because many large algae, the bodies and excretion of marine animals were observed in tidal flat^18^. The previous study results about the distribution and accumulation of phosphorus and organic matter of the sediment in the estuary^19^,^39^ can well explain why the soil TP content was relative stable and the significant relation of TP and land formation age was not observed in present study (Fig. 5).

### Soil salinity

The soil salinity not only was a key factor which decided natural vegetation distribution in coastal wetlands^28^,^35^, but also increased rates of net N and P mineralization fluxes and turnover in tidal wetland soils^55^,^56^, resulting in alteration of the soil nutrient content and distribution. In order to improve the soil quality to be suitable for farming, the techniques of salt leaching and salt reducing were applied in cropland of the YRD like other saline land in some countries^57^,^58^,^59^,^60^. Therefore, the soil salinity in croplands and paddy fields were much higher than those in natural wetlands (Fig. 4F) and soil salinity tend to increase with decreasing land formation age from 1855 to present (Fig. 5). The high soil salinity pattern with low N content in study region appeared in degraded wetland in northern part because of seawater intrusion, new-born wetland in eastern part and central part of which dominant land uses were residential land cropland changed from cultural pond (Fig. 4A–C,E). There were significant negative relations of soil salinity and TOC and TN in study region, while the significant relation of nutrients and pH was not observed (Table 2). The soil salinity not only was a key factor which decided natural vegetation distribution in coastal wetlands^25^,^28^,^35^, but also increased rates of net N and P mineralization fluxes and turnover in tidal wetland soils^55^, resulting in alteration of the soil nutrient content and distribution. Previous study showed that there were significant positive relations between soil salinity and TOC and TN (*p* < 0.05) in new-born wetland of the YRD^13^,^18^. To agree with this point, the similar relationships also observed in this study at same part of study region. The opposite relations of soil TOC, TN with salinity between new-born wetland region and whole study region reflected that the pressure of anthropogenic activities in the YRD were greatly heavy and had altered the original distribution regulations of nutrients and salinity in natural wetlands.

## Conclusions

The Landsat Thematic Mapper digital images with ground resolution of 30 meters of 2009 were used to study the lands use and land cover and variations in TOC, TN, NH_4_^+^-N, NO_3_^−^-N and TP content were evaluated in soil samples collected from 89 soil sampling sites of the modern YRD, eastern China, during 2009–2012 in this study. Results revealed that TOC, TN, NH_4_^+^-N, and NO_3_^−^-N contents in soils varied systematically with depth in coastal zone, with highest N and TOC contents found in land use of cropland in southern part of the YRD and lowest N and TOC contents found in natural wetland of *Suaeda* wetland. Although the TP content in 0–10 cm soil layer is slightly higher than that in 10–30 cm, TP content was relatively stable in profiles and in different land uses. Higher contents of TOC and N were observed in soils of older formation age land in whole study region, while the regulation is opposite in the new-born natural wetland part. The study results indicate that the anthropogenic activities can greatly alter the nutrient distribution pattern by change the land use in the estuary wetland and highlight the land use, land formation age and soil salinity were important factors to influence the nutrient distribution and variations in coastal wetlands of the Modern YRD.

## Additional Information

**How to cite this article**: Yu, J. *et al*. Distribution of carbon, nitrogen and phosphorus in coastal wetland soil related land use in the Modern Yellow River Delta. *Sci. Rep.* **6**, 37940; doi: 10.1038/srep37940 (2016).

**Publisher's note:** Springer Nature remains neutral with regard to jurisdictional claims in published maps and institutional affiliations.

## Figures and Tables

**Table 1:** Statistical results of nitrogen, phosphorus and TOC contents of 0–30 cm soil layer in the study area (*n* = 4179, Aver., average value; s. d., Standard deviation; Max., maximum value; Min., minimum value; Med., median value).

	soil layer (cm)	TOC (g kg^−1^)	NO_3_^—^N (mg kg^−1^)	NH_4_^+^-N (mg kg^−1^)	TN (mg kg^−1^)	TP (mg kg^−1^)
Aver.		5.31	7.03	8.19	460.01	615.33
s. d.		2.74	12.34	6.91	254.43	96.21
Max.	0–10	20.23	60.95	44.45	1442.79	1023.41
Min.		1.69	0.33	1.05	122.40	439.52
Med.		4.80	2.60	5.80	415.44	607.28
Aver.		3.99	4.81	6.86	362.02	579.14
s. d.		2.14	8.36	5.25	186.00	90.32
Max.	10–20	12.54	56.05	29.95	1314.58	932.68
Min.		0.85	0.25	0.55	70.46	281.09
Med.		3.52	2.05	5.40	329.79	573.34
Aver.	20–30	3.30	4.19	6.52	302.40	578.68
s. d.		1.77	6.99	4.05	156.72	81.76
Max.		9.17	38.25	18.10	762.15	867.16
Min.		0.69	0.41	0.25	85.56	406.08
MED.		2.89	2.00	5.45	250.68	564.87
Aver.		4.21	5.36	7.20	375.91	591.27
s. d.		2.40	9.59	5.58	213.44	91.16
Max.	0–30	20.23	60.95	44.45	1442.79	1023.41
Min.		0.69	0.25	0.25	70.46	281.09
Med.		3.77	2.15	5.57	324.30	579.64

**Table 2:** Matrix of correlation coefficient between salinity, pH values and nutrient contents in the Yellow River Delta.

	EC	pH	TOC	NO_3_^−^-N	NH_4_^+^-N	TN	TP
EC	1						
pH	−0.024	1					
TOC	−0.094*	−0.032	1				
NO_3_^−^-N	−0.078	−0.045	0.193**	1			
NH_4_^+^-N	−0.039	−0.025	0.524**	0.390**	1		
TN	−0.119**	−0.033	0.815**	0.304**	0.583**	1	
TP	0.040	0.044	−0.084	−0.096*	−0.092*	−0.083*	1

^*^Correlation is significant at the 0.01 level (2-tailed).

^**^Correlation is significant at the 0.05 level (2-tailed).
